# Molecular Dynamics and Solvated Interaction Energy Prioritize Cannabidiol and Cannabinol as Variant-Spanning SARS-CoV-2 RBD–ACE2 Interface Blockers

**DOI:** 10.3390/molecules31081253

**Published:** 2026-04-10

**Authors:** Napat Kongtaworn, Silpsiri Sinsulpsiri, Chonnikan Hanpaibool, Phornphimon Maitarad, Panupong Mahalapbutr, Thanyada Rungrotmongkol

**Affiliations:** 1 Program in Bioinformatics and Computational Biology, College of Interdisciplinary and Integrative Studies, Chulalongkorn University, Bangkok 10330, Thailand; 2 Center of Excellence in Biocatalyst and Sustainable Biotechnology, Department of Biochemistry, Faculty of Science, Chulalongkorn University, Bangkok 10330, Thailand; 3 Research Center of Nano Science and Technology, Shanghai University, Shanghai 200444, China; 4 Emerging Industries Institute, Shanghai University, Jiaxing 314006, China; 5 Department of Biochemistry, Center for Translational Medicine, Faculty of Medicine, Khon Kaen University, Khon Kaen 40002, Thailand

**Keywords:** SARS-CoV-2, spike RBD–ACE2 interface, cannabinoids, cannabidiol, cannabinol, molecular dynamics simulations, MM/GBSA, solvated interaction energy (SIE)

## Abstract

Severe acute respiratory syndrome coronavirus 2 (SARS-CoV-2) enters host cells when the spike receptor-binding domain (RBD) engages angiotensin-converting enzyme 2 (ACE2). Cannabinoid scaffolds have recently been reported to bind S1/RBD, block spike-mediated membrane fusion, and modulate host inflammatory pathways, making them attractive candidates for entry inhibition. Here, we applied an integrated computational pipeline to prioritize cannabis-derived compounds as interfacial blockers of the RBD–ACE2 complex across variants. Eleven phytocannabinoids were docked into the wild-type (WT) RBD–ACE2 interface, identifying three cavities, with ligands preferentially occupying pocket 1. Complexes were subjected to triplicate 200 ns all-atom molecular dynamics (MD) simulations for WT, Delta, and Omicron BA.1 RBD–ACE2. Binding energetics were quantified using molecular mechanics/generalized Born surface area (MM/GBSA) and solvated interaction energy (SIE), and per-residue contributions were analyzed together with solvent-accessible surface area (SASA) and residue interaction networks. Among all compounds, cannabidiol (CBD) and cannabinol (CBN) were the only ligands that remained stably bound in pocket 1 for all variants. CBN showed the most favorable ligand–complex binding in WT, whereas CBD preserved favorable binding in Omicron BA.1 despite reduced interface burial, indicating that van der Waals/electrostatic complementarity and solvation, rather than surface coverage alone, govern affinity. Both ligands weakened modeled RBD–ACE2 binding by perturbing hot-spot residues centered on Y505 or N501Y in RBD and E37, A387, and R393 in ACE2. Overall, our results highlight CBD and CBN as tractable, variant-spanning interface disruptors and illustrate how MD-based free-energy calculations can support computational drug discovery against evolving viral protein–protein interfaces.

## 1. Introduction

**Figure 1:**
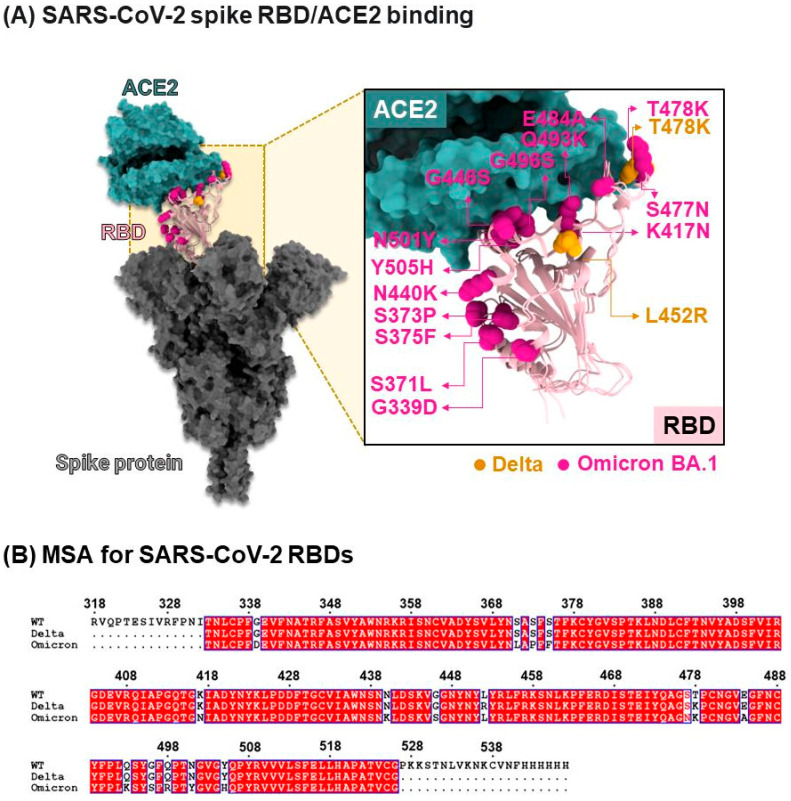
(**A**) Crystal structure of the spike receptor binding domain (RBD) in complex with ACE2 receptor (PDB ID: 7A94) [7]. (**B**) Sequence alignment of the wild-type (WT), Delta, and Omicron BA.1 variants of the RBD. Dots indicate gaps in the sequence alignment.

The emergence of highly pathogenic coronaviruses, such as severe acute respiratory syndrome coronavirus (SARS-CoV) and Middle East respiratory syndrome coronavirus (MERS-CoV), has led to severe respiratory and intestinal infections in humans [1]. In 2019, a new coronavirus, severe acute respiratory syndrome coronavirus 2 (SARS-CoV-2), was identified, quickly spreading worldwide and subsequently declared a global pandemic by the World Health Organization (WHO) [2]. The SARS-CoV-2 genome is a positive-sense single-stranded RNA (+ssRNA) approximately 30 kb in length, encoding the nucleocapsid (N), membrane (M), envelope (E), and spike (S) proteins [3]. Among these, the S protein plays a crucial role in viral entry into host cells through membrane fusion [4]. It consists of two subunits, S1 and S2 (Figure 1), with S1 containing the receptor-binding domain (RBD) responsible for receptor interaction during fusion, while S2 maintains the stability of the prefusion state [5]. Specifically, the RBD of SARS-CoV-2 interacts with the angiotensin-converting enzyme 2 (ACE2) receptor present on human cells, facilitating viral entry into host cells [6].

Mutations occurring in the RBD of the S protein have been found to significantly enhance the binding affinity between the RBD and ACE2 [8]. In addition, other spike mutations outside the RBD, such as D614G, have also been reported to increase viral infectivity [9]. In 2020, a mutation known as N501Y emerged within the RBD binding site of the S protein of SARS-CoV-2, leading to its rapid spread in England, South Africa, and Brazil [10,11]. The Global Initiative on Sharing Avian Influenza Data [12] has classified the N501Y mutation into distinct variants in different regions. The N501Y mutation found in England is designated as 20I (20I/501Y.V1, Alpha), while the mutations observed in South Africa and Brazil are classified as 20H (20H/501Y.V2, Beta) and 20J (20J/501Y.V3, Gamma), respectively. The Alpha variant (20I/501Y.V1) exhibits mutations involving N501Y and deletions at residues 69 and 70, which contribute to immune evasion. On the other hand, strains originating from South Africa and Brazil, namely N501Y.V2 and N501Y.V3, demonstrate reduced neutralization by convalescent plasma from previously infected individuals compared to other strains [13]. Furthermore, in late 2021, the WHO identified two Variants of Concern: 21A (Delta) and a more recent strain named Omicron with sublineages like BA.1, BA.2, BA.4, BA.5, XBB, and XBB1.5. Both variants possess multiple mutations in the RBD, and experimental studies suggest that Omicron, in particular, displays enhanced infectivity due to its increased ability to evade human antibodies [14]. In 2025, Omicron sublineages continue to circulate globally and contribute to ongoing transmission dynamics.

Drug development is important to prevent novel mutant SARS-CoV-2 entry into host cells. To block SARS-CoV-2 in the host cell [15], several compounds and drugs have been screened within the protein–protein interface of the RBD/ACE2 complex [16]. The previous docking results suggested that streptomycin, ciprofloxacin, and glycyrrhizic acid may inhibit spike SARS-CoV-2 [17]. Moreover, biomedical compounds, e.g., highly concentrated cannabidiol (CBD) and mixtures of CBD, can down-regulate ACE2 expression in the heart, blood vessels, gut, thyroid, adipose tissue, and lungs [18].

CBD can inhibit infection of early-stage SARS-CoV-2 in vitro and in vivo [19]. In addition, CBD mitigates spike-induced epithelial cytotoxicity and hyperinflammation in Caco-2 cells—via PPARγ-dependent suppression of TLR4/NLRP3/Caspase-1—while restoring barrier integrity (TEER) and tight junctions, indicating host-protective anti-inflammatory effects relevant to mucosal tissues [20]. In addition, the experimental study suggested that the cannabis compounds can interact with the RBD. Among these, cannabigerolic acid (CBGA) and cannabidiolic acid (CBDA) have a high potential to block viral fusion compared with other cannabis compounds in Alpha and Beta strains using magnetic microbead affinity selection screening (MagMASS) [21]. In addition, CBDA and CBGA bind the RBD with micromolar affinity and prevent cellular entry by both pseudovirus and live virus, including early variants of concern, thereby supporting an RBD-centric entry blockade [21]. In parallel, cannabigerol (CBG) and cannabicyclol (CBL) reduce pseudotyped-virus entry, lower infectious titers, and directly inhibit spike-mediated membrane fusion in cell–cell fusion assays, highlighting a fusion-level mechanism that complements receptor recognition [22]. Beyond entry, CBD potently inhibits the viral main protease (M^pro^; low-micromolar IC50) and moderately inhibits ACE2, with Δ9-THC being comparatively weaker and CBN less active in those assays, suggesting potential dual action on replication and entry [23]. Moreover, molecular dynamics (MD) simulation indicated that cannabinolic acid and cannabigerovarinic acid can interact with the binding site between ACE2 and RBD of spike SARS-CoV-2 [24]. CBD and Cannabivarin (CVN) were docked to the binding sites of WT ACE2, TMPRSS2, NRP1, and interleukin (IL)-6, and the complexes were simulated for 100 ns. The binding results indicated that the binding affinity between both compounds and ACE2 was stronger than the binding affinity between both compounds and other proteins, and CBD/ACE2 shows the strongest binding affinity compared to CVN/ACE2 [25].

**Figure 2:**
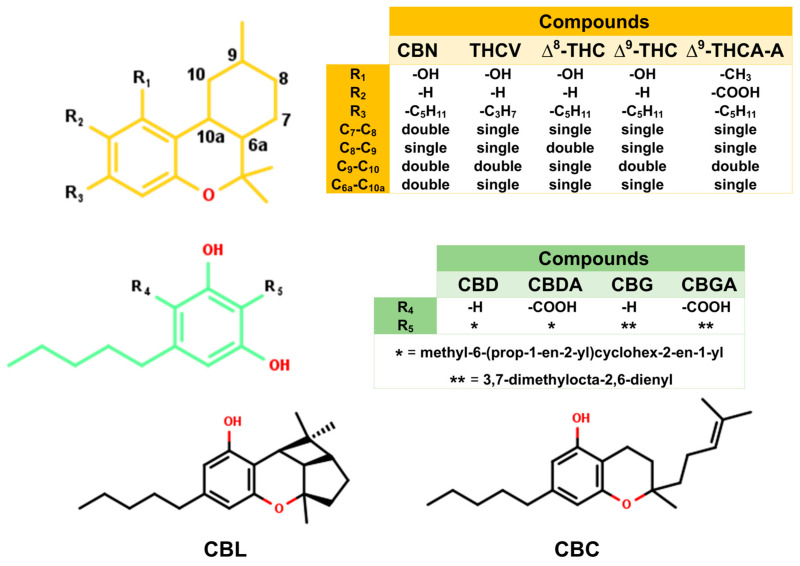
The 2D structures of 11 cannabis compounds that were docked to the binding site of the WT RBD and ACE2 interface, including ∆^8^-tetrahydrocannabinol (∆^8^-THC), ∆^9^-tetrahydrocannabinol (∆^9^-THC), cannabigerolic acid (CBGA), cannabidiolic acid (CBDA), cannabichromene (CBC), cannabidiol (CBD), cannabigerol (CBG), cannabinol (CBN), cannabicyclol (CBL), ∆^9^-tetrahydrocannabinolic acid A (∆^9^-THCA-A), and tetrahydrocannabivarin (THCV). In the inset tables, * denotes methyl-6-(prop-1-en-2-yl)cyclohex-2-en-1-yl and ** denotes 3,7-dimethylocta-2,6-dienyl.

In this work, 11 cannabis compounds (Figure 2) were investigated to identify candidate compounds that can prevent viral fusion by blocking the binding between the SARS-CoV-2 wild-type (WT)/mutant RBD and ACE2. To model the cannabis compounds and their blocking of the RBD/ACE2 complex, the cannabis compounds were docked with the WT, Delta, and Omicron strains of the RBD and human ACE2. Then, their structural and energetic properties were investigated using molecular dynamics (MD) simulations and binding energy calculations based on the molecular mechanics/generalized Born surface area (MM/GBSA) and solvated interaction energy (SIE) methods [26,27]. This work helps clarify the mechanism by which cannabis compounds block viral fusion, which can assist the development of novel vaccines and drugs that block SARS-CoV-2 entry.

## 2. Results and Discussion

### 2.1. Molecular Docking of Cannabis Compounds Within the RBD/ACE2 Binding Pocket

**Figure 3:**
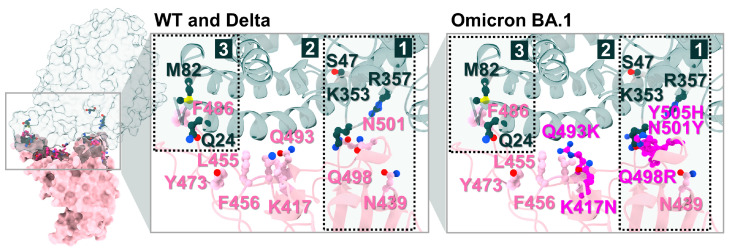
Binding interface and key residue mapping between the SARS-CoV-2 WT RBD (pink) and the human ACE2 receptor (green). Three major interaction pockets (labeled 1, 2, and 3) were identified as potential targets for small-molecule inhibition [28], and they are shown together with key interface residues and selected Omicron BA.1 mutation sites relevant to the pocket regions. WT and Delta are shown together, where the labeled interface residues are conserved in the present structural representation. Key interface residues and selected mutation sites are shown as ball and stick representations in both panels. Atom colors follow standard element coloring (e.g., O, red; N, blue). The full variant-specific mutation map is provided in Figure 1B.

**Figure 4:**
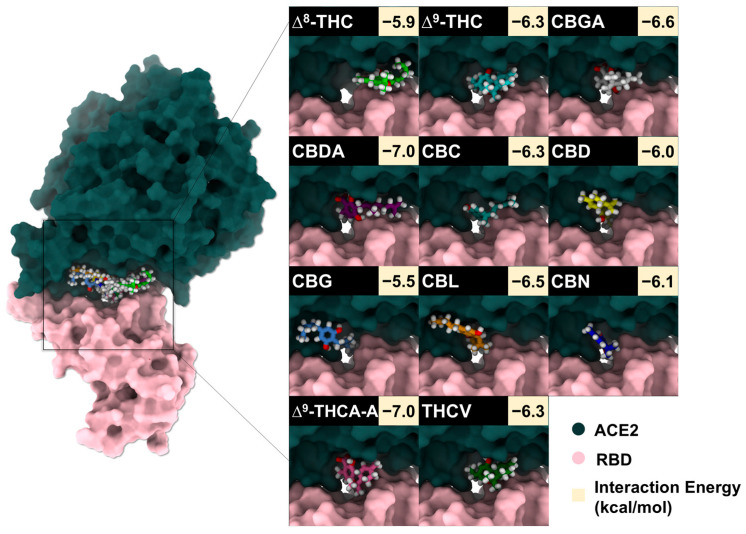
Surface representation of the WT SARS-CoV-2 RBD (pink) and ACE2 (green) interface showing the docked poses of 11 cannabis compounds within pockets 1–3. Insets show representative binding conformations and their corresponding interaction energies for each compound.

To identify candidate compounds capable of disrupting the interaction between the SARS-CoV-2 spike RBD and ACE2, 11 cannabis-derived compounds—including ∆^8^-THC, ∆^9^-THC, CBGA, CBDA, CBC, CBD, CBG, CBN, CBL, ∆^9^-THCA-A, and THCV—were docked into the RBD/ACE2 interface (see Figure 2 for compound structures). Based on the electrostatic distribution of charged and polar residues, the RBD–ACE2 interface could be partitioned into three interaction regions (pockets 1, 2, and 3) (Figure 3), consistent with the cluster-based interface description reported previously [28]. These regions represent hot spots involved in stabilizing the RBD–ACE2 interaction and serve as plausible targets for small-molecule inhibition [28]. As shown in Figure 4, all 11 cannabis compounds docked preferentially into pockets 1 and 2, with no compounds binding primarily to pocket 3. Their calculated interaction energies ranged from −5.5 to −7.0 kcal/mol, suggesting moderate binding affinities. Notably, CBDA and ∆^9^-THCA-A exhibited the most favorable docking scores (−7.0 kcal/mol), followed closely by CBGA (−6.6 kcal/mol), CBL (−6.5 kcal/mol), and CBN (−6.1 kcal/mol). This binding profile implies that acidic cannabinoids (e.g., CBDA and THCA-A) may establish more stabilizing interactions with the polar interface residues. The docked conformations also reveal that many compounds span across residues from both the RBD (pink) and ACE2 (green), suggesting potential for interface blockade. These docked complexes were subsequently used as starting structures for molecular dynamics (MD) simulations, enabling further evaluation of their dynamic stability and interaction persistence under physiological conditions. Together, these docking results support the hypothesis that specific phytocannabinoids can effectively occupy critical interface pockets of the RBD/ACE2 complex, with potential to sterically hinder or allosterically modulate spike–receptor recognition.

### 2.2. Efficiency of Cannabis Compounds Blocking WT RBD/ACE2 Complex

**Figure 5:**
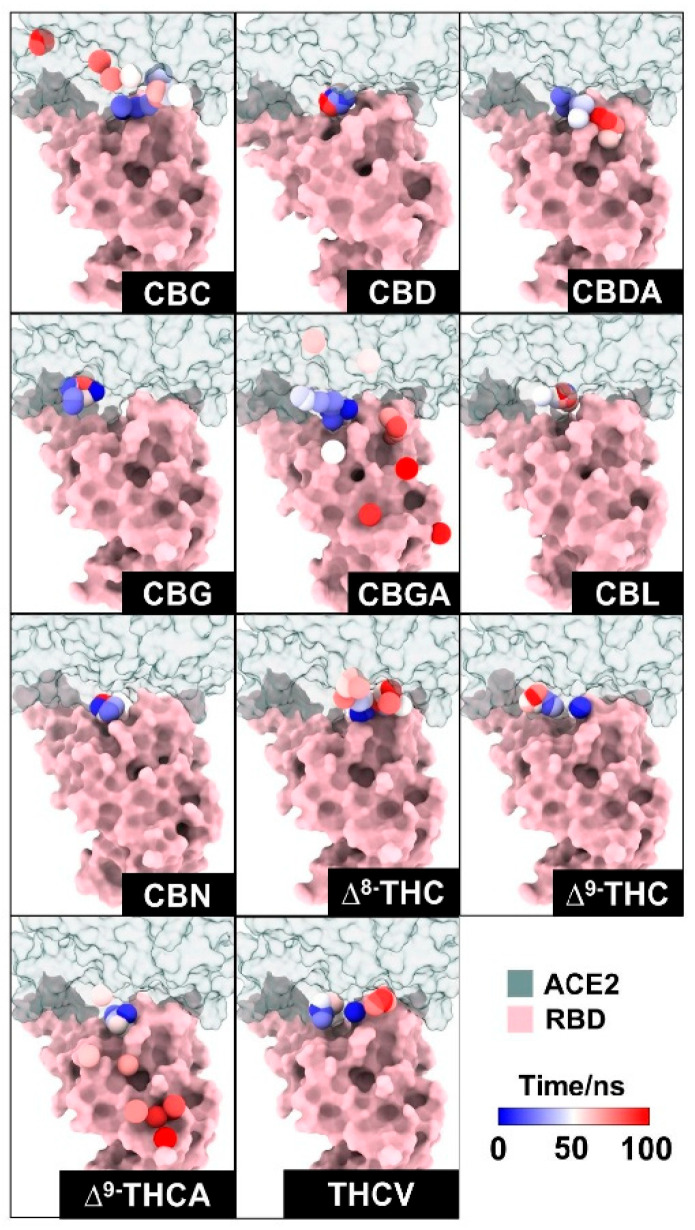
Dynamics of 11 cannabis compounds in the WT RBD (pink) and ACE2 (green) complex. The center of mass of each compound is presented as a dot, and the color of the dot represents the simulation time, ranging from 0 (the docking position) to 100 ns (blue to red).

The movement of each compound during the MD simulations is presented in Figure 5. The red points represent the center of mass of each compound moving around the RBD and ACE2 surface. Results revealed that CBC, CBGA, and ∆^9^-THCA-A moved out of the binding pocket of RBD/ACE2. In addition, CBDA moved slightly out from the binding pocket of the protein complex to bind with the RBD, whereas ∆^8^-THC, ∆^9^-THC, and THCV moved slightly away from the binding pocket of the protein complex to bind with ACE2. CBG and CBL can interact with residues within the binding complex during 100 ns; however, after 100 ns, the increased distances of CBG and CBL (Figure S1) represented the dissociation of compounds from the initial position or binding site of the protein complex. The results indicated that both compounds moved away from the binding pocket of the protein complex. Notably, only CBD and CBN can interact with residues in the binding pocket during 100 ns, and both compounds still interacted within the binding pocket of RBD/ACE2 after 200 ns.

Previously, the cannabis compounds were screened and ranked by binding affinity to the spike protein using MagMASS [21]. The highest binding affinities for the spike protein are CBDA and CBGA [21]. Moreover, infection inhibition assay results suggested that CBDA and CBGA could block SARS-CoV-2 entry into human epithelial cells [21]. In our case, CBDA and CBGA can be docked in the binding pocket, but during 100 ns of simulation, CBDA and CBGA moved out of the binding pocket of the RBD/ACE2 complex. Our findings suggested that CBD and CBN might prevent SARS-CoV-2 from entering human cells by blocking the binding between the RBD and ACE2.

**Figure 6:**
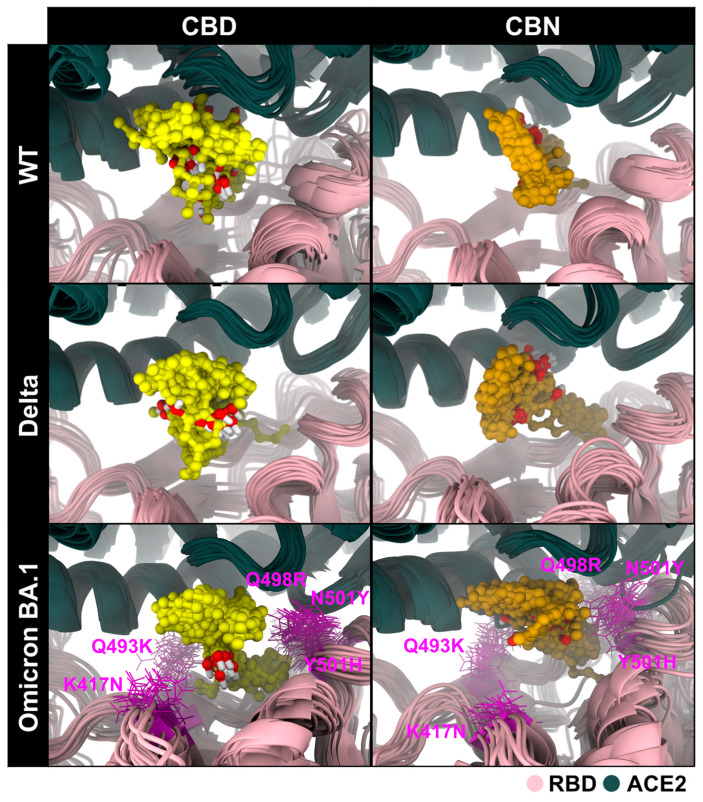
Structural superimpositions of CBD and CBN within binding pocket 1 of WT, Delta, and Omicron BA.1 RBD/ACE2 during 190–200 ns of simulation. Selected pocket-proximal Omicron BA.1 mutated residues visible in the current view are annotated to provide structural context for the SASA differences summarized in Table 2. CBD and CBN are shown as yellow and orange sphere representations in the current view.

Overall, only cannabidiol (CBD) and cannabinol (CBN) were selected for further study against the WT, Delta, and Omicron BA.1 RBD–ACE2 systems. CBD-bound WT, Delta, and Omicron BA.1 RBD–ACE2 complexes (CBD/WT RBD–ACE2, CBD/Delta RBD–ACE2, and CBD/Omicron BA.1 RBD–ACE2) and CBN-bound WT, Delta, and Omicron BA.1 RBD–ACE2 complexes (CBN/WT RBD–ACE2, CBN/Delta RBD–ACE2, and CBN/Omicron BA.1 RBD–ACE2) were each subjected to three independent 200 ns all-atom molecular dynamics simulations. The stabilities of all combined systems were plotted and shown in Figure S2. After that, the three replicates of each system were combined into one trajectory, and the combined trajectory from 190 ns to 200 ns (i.e., the last 10 ns of simulation) was collected to investigate and compare the interactions and binding affinities of all systems. The structural superimposition over the last 10 ns of simulation revealed that CBD and CBN interacted with three strains of RBD/ACE2 in the binding pocket 1, as shown in Figure 6. However, the structure of CBN was more stable than that of CBD because the aromatic ring of CBD bonded with R_5_ (-methyl-6-(prop-1-en-2-yl)cyclohex-2-en-1-yl, Figure 2) was more twisted, in good agreement with the binding free energy calculation based on the SIE method (Table 1).

Binding free energy ($∆G_{bind}$) and solvent-accessible surface area (SASA, in Table 2) analyses collectively provide a detailed picture of the molecular interactions between cannabis compounds and the RBD/ACE2 interface across SARS-CoV-2 variants. To provide structural context for these system-level SASA differences, selected pocket-proximal Omicron BA.1 mutated residues are annotated in Figure 6. For the WT complex, CBN demonstrated the most favorable $∆G_{bind}$ (−6.69 ± 0.03 kcal/mol), along with the largest %SASA reduction (52.1%), indicating strong binding and substantial coverage of the RBD/ACE2 interface. CBD also induced a notable %SASA reduction (35.0%) in WT, though to a lesser extent than CBN, consistent with its slightly weaker binding energy (−6.09 ± 0.08 kcal/mol). In the Delta system, both compounds showed more modest %SASA reductions (CBD: 36.3%, CBN: 34.5%), along with correspondingly reduced binding energies, suggesting less extensive interface burial compared to WT. Interestingly, for Omicron BA.1, although both compounds exhibited the lowest %SASA values (CBD: 26.6%, CBN: 31.0%), their $∆G_{bind}$ values remained comparable to or better than those in Delta, implying that favorable interactions may still occur in a more confined or conformationally restricted binding interface.

These findings suggest that while %SASA reduction provides insight into how much of the protein–protein interface is physically shielded by the ligand, it does not always directly correlate with binding affinity. For example, CBD in Omicron BA.1 showed the lowest %SASA (26.6%) yet retained a favorable $∆G_{bind}$ (−6.06 ± 0.22 kcal/mol), likely due to strong electrostatic contributions ($∆E_{c}$ = −10.23 ± 0.77 kcal/mol). Conversely, CBN’s consistently large van der Waals (vdW) contributions (e.g., −40.01 ± 0.22 kcal/mol for WT) appear to drive both extensive interface masking and strong binding. This interplay highlights that effective binding involves not only surface occlusion but also optimal energetic complementarity.

### 2.3. Pharmacophore and Interaction Profiles of CBD and CBN at RBD/ACE2 Interface

**Figure 7:**
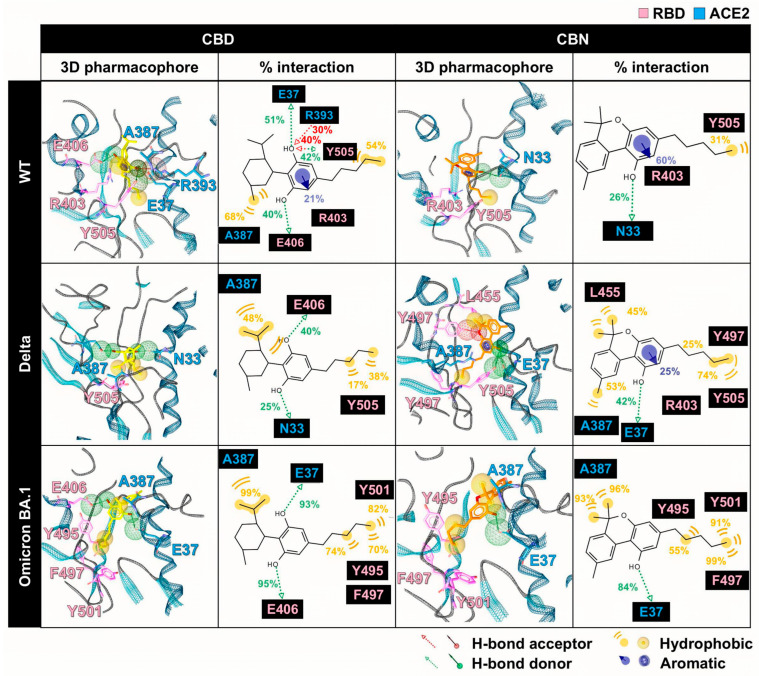
Pharmacophore analysis between cannabis compounds (CBD and CBN) and RBD/ACE2 complex using LigandScout v4.4.2. The ACE2 and RBD residues are labeled in green and pink, respectively.

The 3D/2D pharmacophores and interaction frequencies (% interaction) between CBD/CBN and the WT/mutant RBD/ACE2 complexes were generated using LigandScout v4.4.2 (Inte:Ligand GmbH, Vienna, Austria) (Figure 7). To capture ensemble behavior, the interaction frequencies were computed across MD snapshots from the equilibrated analysis window (190–200 ns; 1000 frames per system) and summarized over the triplicate simulations. For visualization, a representative snapshot from this window was selected, and all complexes were displayed in a consistent orientation to enable direct comparison; variant labels were cross-checked, and mutated residues were annotated in the structural panels.

**Figure 8:**
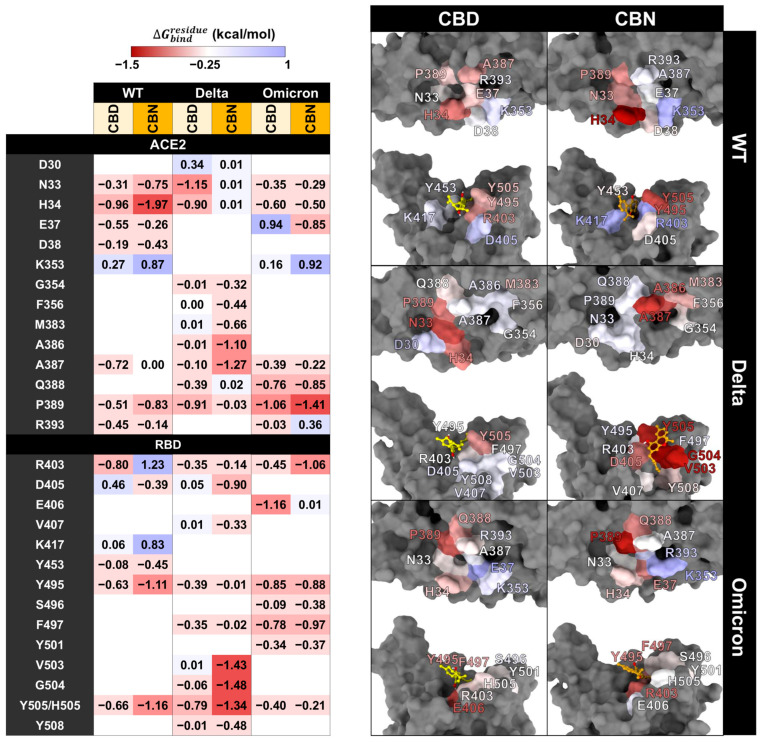
${\Delta G}_{bind}^{residue}$ between the cannabis compound and the RBD/ACE2 complex is presented on the left, and the structure of the cannabis compound interacting with RBD/ACE2 is presented on the right. Color labels: ${\Delta G}_{bind}^{residue}$ ranges from red (−1.5 kcal/mol) to blue (1.00 kcal/mol).

However, the pharmacophore result can partially reveal the binding pattern and identify the intermolecular interactions of cannabis compounds and RBD/ACE2. So, to clarify the interaction of CBD and CBN with the RBD/ACE2 complex, the key binding was evaluated using the ${\Delta G}_{bind}^{residue}$ calculation based on the MM/GBSA method (Figure 8). In this work, the energetic stabilization and destabilization between RBD and ACE2 were considered within the ranges of ≤−0.30 and ≥0.30 kcal/mol, respectively. As in the case of the WT system, N33, H34, E37, A387, P389, and R393 in ACE2 (−0.31, −0.96, −0.55, −0.72, −0.51, and −0.45 kcal/mol) and R403, Y495, and Y505 in RBD (−0.80, −0.63, and −0.66 kcal/mol) showed strong binding energy to CBD. The E37, A387, and R393 in ACE2 and R403 and Y505 in RBD were also matched with the pharmacophore results. The CBN presented the same critical binding residue as CBD, but some residues showed more stabilization, including N33, H34, D38, and P389 in ACE2 and D405, Y453, Y495, and Y505 in RBD. The results indicate that the binding affinity of CBN to the WT RBD/ACE2 complex is higher than that of CBD. Representative per-residue decomposition profiles of CBD and CBN in the WT, Delta, and Omicron BA.1 RBD/ACE2 complexes are shown in Figure 8.

In the Delta and Omicron BA.1 systems, most of the stabilization residues that interacted with CBD are similar to the WT system (N33, H34, and P389 in ACE2 and R403, Y495, and Y505 in RBD). Only two stabilization residues (F497 and Q388) were detected in Delta and Omicron BA.1 systems. The residue A387 interacted with CBD in the WT and Omicron BA.1 systems. In contrast, there are a few destabilization residues that interacted with CBD (D30 in Delta and E37 in Omicron BA.1). In the CBN/Delta RBD/ACE2 case, most stabilization residues are different (V407, V503, G504, Y508, G354, F356, M383, and A386) and only three stabilization residues (A387, D405, and Y505) are similar to those for CBN/WT RBD/ACE2. In CBN/Omicron BA.1 RBD/ACE2, three stabilization residues, H34, P389, and Y495, are similar to those of CBN/WT RBD/ACE2, and six stabilization residues, E37, Q388, P389, S496, F497, and Y501, are different from those of CBN/WT RBD/ACE2. Pattern of stabilization/destabilization residues of RBD/ACE2 interacted with CBD in WT, Delta, and Omicron BA.1 systems showed similar trend, while that with CBN in Delta system is different to that in WT and Omicron BA.1. The patterns of stabilization/destabilization residues between CBN and ACE2 in Omicron BA.1 are similar to that in WT, while those between CBN and RBD in Omicron BA.1 are different from the pattern between CBN and ACE2 in WT and Delta.

To estimate the binding affinity of cannabis compounds and RBD/ACE2 complexes, the binding free energies (∆*G_bind_*) of all systems were calculated using the SIE method (Table 1). Results showed that the binding affinity of CBN with the WT, Delta, and Omicron BA.1 systems (∆*G_bind_* = −6.69 ± 0.03, −6.41 ± 0.03, and −6.61 ± 0.05 kcal/mol, respectively) is stronger than that of CBD (∆*G_bind_* = −6.09 ± 0.08, −5.91 ± 0.07, and − 6.06 ± 0.22 kcal/mol, respectively). For the WT system, the ∆*E_vdW_* values of CBD and CBN are −32.84 ± 0.34 and −40.01 ± 0.22 kcal/mol, respectively; for Delta; the ∆*E_vdW_* values of CBD and CBN are −32.56 ± 0.60 and −34.49 ± 0.28 kcal/mol, respectively; and for Omicron BA.1; the ∆*E_vdW_* values of CBD and CBN are −35.84 ± 1.00 and −38.74 ± 0.34 kcal/mol, respectively. Moreover, the coulombic energy (∆*E_c_*) showed a weak interaction energy compared with ∆*E_vdW_*. The ∆*E_c_* values between CBD and the WT, Delta, and Omicron BA.1 strains are −8.59 ± 0.42, −6.65 ± 0.33, and −10.23 ± 0.77 kcal/mol, respectively. The ∆*E_c_* values between CBN and the WT, Delta, and Omicron BA.1 strains are −4.61 ± 0.12, −1.6 ± 0.11, and −8.99 ± 0.26 kcal/mol, respectively.

From the pharmacophore analysis, key binding residue results, and SIE calculations, our results suggested that CBD and CBN could bind to WT and mutant RBD/ACE2 complexes. The CBD conformation is more flexible than the CBN conformation because of the twisted R_5_ in the aromatic ring of CBD. The pharmacophore analysis indicated that CBD and CBN could bind to WT and mutant RBD and ACE2 with the same interaction pattern, such as vdW, H-bond, and aromatic interactions with RBD and ACE2 residues. The strong interactions between cannabis compounds and RBD/ACE2 complexes are vdWs and H-bonds, whereas the aromatic interaction is only found in the WT system and CBN/Delta RBD/ACE2. The interactions of all systems were supported by the vital binding residue resulting from MM/GBSA calculations. The SIE calculation result suggested that the binding affinity of CBN is stronger than that of CBD. Moreover, the vdW is the main interaction that can promote the binding affinity between cannabis compounds and RBD/ACE2 complexes. Several natural compounds were found to bind to pocket 1 of the RBD/ACE2 complex, such as rutin, quercitrin, theaflavin 3,3′-digallate, procyanidin A1, procyanidin B2, and procyanidin [29]. These natural compounds can interact with Y505 by π-π stacking and H-bonding, and the same is true for E406 by H-bonding, similar to CBD and CBN systems. Both compounds can interact with Y505 (vdW) and E406 (H-bond) [29].

### 2.4. Effects of CBD and CBN on the Binding Affinity Between the WT, Delta, and Omicron BA.1 RBD and the ACE2

**Figure 9:**
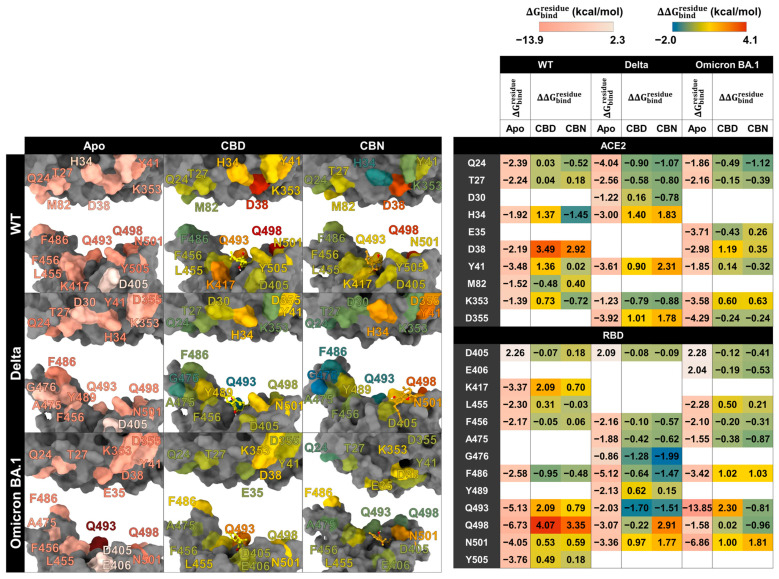
DC calculation between RBD and ACE2. ${\Delta G}_{bind}^{residue}$ between RBD and ACE2 (**left**); structure of RBD and ACE2 (**right**). Colors labels: ${\Delta G}_{bind}^{residue}$ ranges from red (−13.90 kcal/mol) to white (2.30 kcal/mol); ${\Delta \Delta G}_{bind}^{residue}$ ranges from blue (−2.00 kcal/mol) to red (4.10 kcal/mol). Residues in the structural panels and the table are color-coded according to the corresponding ${\Delta G}_{bind}^{residue}$ and ${\Delta \Delta G}_{bind}^{residue}$ values.

The key binding residues between RBD and ACE2 were evaluated using the MM/GBSA (Figure 9). In this work, the energetic stabilization and destabilization between RBD and ACE2 were collected within the ranges of ≤−2 and ≥2 kcal/mol, respectively. Residues were first selected when at least one system showed an absolute dc value of ≥2 kcal/mol at that position (i.e., dc ≤ −2 kcal/mol for stabilization or dc ≥ 2 kcal/mol for destabilization). After this residue-level selection, the corresponding dc values from the other systems at the same residue were also reported for cross-system comparison, even when their magnitudes were below 2 kcal/mol. The number of destabilization residues from ${\Delta \Delta G}_{bind}^{residue}$ can help identify the decrease in the key binding affinity of the RBD/ACE2 complex when the compounds interact with the binding pocket of the complex. In WT systems, after CBD interacts with the protein complex, we found that there are ten destabilization residues of WT RBD associated with ACE2 (K417, L455, Q493, Q498, N501, and Y505 in RBD and H34, D38, Y41, and K353 in ACE2), but there are two stabilization residues (F486 in RBD and M82 in ACE2). For the CBN system, there are eight destabilization residues of WT RBD associated with ACE2 (D405, K417, Q493, Q498, N501, and Y505 in RBD and T27, D38, and M82 in ACE2), but there are three stabilization residues (L486 in RBD and Q24 and H34 in ACE2). Moreover, the ${\Delta \Delta G}_{bind}^{residue}$ values of CBD exhibit dramatic decreases in the binding energies of K417, Q493, Q498, H34, D38, and Y41 ${(\Delta \Delta G}_{bind}^{residue}$ = 2.09, 2.09, 4.07, 1.37, 3.49, and 1.36 kcal/mol, respectively), and values of CBN exhibit significant decreases in the binding energies of Q498 and D38 ${(\Delta \Delta G}_{bind}^{residue}$ = 3.35 and 2.92 kcal/mol, respectively).

In Delta systems, CBD and CBN can destabilize the Y489, N501, and Y505 residues in RBD (${\Delta \Delta G}_{bind}^{residue}=$ 0.62, 0.97, and 0.97 kcal/mol, respectively) and H34, Y41, and D355 in ACE2 (${\Delta \Delta G}_{bind}^{residue}=$ 1.40, 0.90, and 1.01 kcal/mol, respectively). For Omicron BA.1 systems, the CBD can destabilize L455, F486, R493, and N501Y in RBD (${\Delta \Delta G}_{bind}^{residue}=0.50,\;1.02,\;2.30,\;and\;1.00$ kcal/mol, respectively) and D38, Y41, and K353 in ACE2 (${\Delta \Delta G}_{bind}^{residue}=0.14,\;1.19,\;and\;0.60$ kcal/mol, respectively) and CBN can destabilize the L455, F486, and Y501 residues in RBD (${\Delta \Delta G}_{bind}^{residue}=0.21,\;1.03,\;and\;1.81$ kcal/mol, respectively) and E35, D38, and K353 in ACE2 (${\Delta \Delta G}_{bind}^{residue}=0.26,\;0.35,\;and\;0.63$ kcal/mol, respectively). In addition to apo-form systems, most stabilization residues in the WT and Delta systems showed a similar pattern of key binding residues (D405, F456, F486, Q493, Q498, N501, and Y505 in RBD and Q24, T27, and Y41 in ACE2). However, in the Omicron BA.1 system, the ${\Delta G}_{bind}^{residue}$ of N501 to Y501 increase from −4.05 to −6.86 kcal/mol, while the ${\Delta G}_{bind}^{residue}$ of Y505 to H505 decrease from −3.76 to −1.13 kcal/mol. Moreover, the N501Y in the Omicron BA.1 system plays an essential role in increasing the stability of the binding pocket, but changing from tyrosine 505 to histidine decreases the binding affinity. The results suggest that CBD and CBN can reduce the binding affinity of WT and mutant RBD hot-spot residues to ACE2 within binding pocket 1, including N501Y.

**Figure 10:**
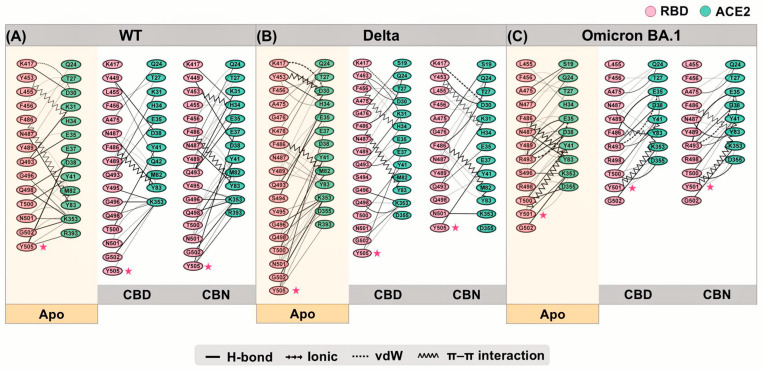
Protein–protein interaction analysis using the RING. (**A**) binding between WT RBD and ACE2, (**B**) Delta RBD and ACE2, and (**C**) Omicron BA.1 and ACE2. Pink asterisks indicate the key hotspot residues highlighted in the interaction-network analysis, particularly Y505 in WT/Delta and N501Y in Omicron BA.1.

The interactions between RBD and ACE2 residues are demonstrated in Figure 10. The starred residues highlight the key hotspot residues emphasized in the present network analysis, particularly Y505 in WT/Delta and N501Y in Omicron BA.1. The residues that showed the energetic destabilization of ≥2 were selected to generate an interaction network using the Residue Interaction Network Generator (RING). Numerous vdW and H-bond interactions were found in the WT, Delta, and Omicron BA.1 systems. Moreover, π-π stacking and ionic interactions were slightly detected in the WT, Delta, and Omicron BA.1 systems. In this section, the primary interaction between cannabis compounds and RBD/ACE2 comes in the form of vdWs and H-bonds. Comparing the interaction between compound–protein and protein–protein, the results indicated that CBD and CBN can prevent the binding affinity of protein complexes via vdWs and H-bonds targeted at Y505 in WT and Delta and N501Y in Omicron BA.1 in binding pocket 1.

The interaction network can represent decreased binding residues between RBD and ACE2. The Y505 in WT can interact with E37, K353, and R393. When CBD interacts with Y505, the interaction between Y505 and ACE2 residues decreases to only K353. Whereas CBN cannot destroy interactions between Y505 and ACE2 residues, the percentage of H-bonds between Y505 and E37 decreases. Moreover, the percentage of interaction between other RBD and ACE2 residues is reduced when cannabis compounds interact with binding pocket 1, such as E35, K417, and F456. In the Delta system, CBD and CBN can decrease the number of interaction residues. The Y505 interacting with E37, K353, and R393 can reduce the interaction network in CBD (Y505 interacting with E37 and K353) and CBN (Y505 interacting with E37) systems. For the Omicron BA.1 system, the CBD and CBN can interact with N501Y. The interaction network of N501Y indicated that a strong interaction between N501Y and K353 was reduced. Furthermore, network-based profiling was generated to identify binding and allosteric interactions of the WT, Delta, and Omicron BA.1 RBD/ACE2 complexes [30]. Q498, N/Y501, and Y505 presented the largest interfacial community [30]. In addition, the N501Y is an essential interfacial community, which increases the stability and strengthens the affinity of the global interaction network [30]. Our findings show that CBD and CBN can interact with N501 and Y505. The key binding residues and interaction network obtained by RING suggested that CBD and CBN can decrease the binding affinity between RBD and ACE2, especially for residues in pocket 1.

Previously, the binding affinities of WT, Delta, and Omicron BA.1 were reported. The binding affinity of the Omicron BA.1 system is stronger than the Delta and WT systems, but another report indicated that the binding affinity of the Omicron BA.1 system is weaker than that of the Delta system [31,32]. The experimental study (ELISA bioassay) and MD simulations of Delta and Omicron BA.1 RBD/ACE2 suggested that the binding affinity of Omicron BA.1 RBD to ACE2 is weaker than that of Delta to ACE2 [32]. This is similar to our results, where the binding free energy calculations between RBD and ACE2 indicated that the binding affinity of the Omicron BA.1 system was weaker than that of the Delta system (Table 3). The ∆*E_vdW_* of apo-form in the WT and mutant systems slightly decreased compared with the CBD and CBN systems. In addition, the ∆*G_bind_* between RBD and ACE2 indicated that the binding affinity of WT (∆*G_bind_* = −14.00 ± 0.08 kcal/mol) decreased when CBD (∆*G_bind_* = −11.60 ± 0.08 kcal/mol) and CBN (∆*G_bind_* = −12.43 ± 0.06 kcal/mol) interact with binding pocket 1. For ∆*E_c_*, the Delta and Omicron BA.1 systems increase more than the WT system because of the enhanced polar charge within the binding interface [32]. The decrease in apo-forms in the WT system was also observed in the Delta (∆*G_bind_* decrease from −15.02 ± 0.09 to −14.32 ± 0.13 kcal/mol in CBD and −13.72 ± 0.11 kcal/mol in CBN) and Omicron BA.1 (∆*G_bind_* decrease from −14.12 ± 0.07 to −11.97 ± 0.08 kcal/mol in CBD and −12.35 ± 0.09 kcal/mol in CBN) systems. The results indicated that CBD and CBN can decrease the binding affinity of RBD and ACE2 complexes.

Comparing the key binding residues, binding interactions, and energies between RBD and ACE2 while considering the apo-form, CBD, and CBN indicated that CBD and CBN can decrease binding affinities between the WT, Delta, and Omicron BA.1 RBD spike proteins and ACE2 receptors. According to the SIE results, CBD and CBN present decreasing energetic stabilization, especially for Y505, and increased protein–protein interaction in the WT system. In the mutant system, the CBD and CBN can reduce binding affinities between both proteins, but the efficiency of both compounds decreases. However, in the Omicron BA.1 strain, both compounds can decrease the interaction of the main key residues, such as N501Y, by vdW interactions. The pentyl group in CBD and CBN (Figure 7) plays the same vital role in blocking binding between proteins and other functional groups that interact by π-π stacking or H-bonds with N501Y [29].

The present docking/MD results, which prioritize CBD and CBN as stable occupants of pocket-1 at the RBD–ACE2 interface across variants, align with experimental reports that cannabinoids can act at multiple nodes of the entry pathway. The RBD-targeting activity of CBDA/CBGA—shown to bind S1/RBD and block entry of pseudovirus and live SARS-CoV-2—supports the plausibility that cannabinoid scaffolds can engage spike recognition surfaces; our pocket-1 pharmacophore partially overlaps the reported RBD contact region and rationalizes the variant-spanning stabilization observed in silico [21]. Moreover, fusion-level inhibition by CBG/CBL provides a mechanistic complement to our interface-blocking model; together with our per-residue energy decomposition, this suggests that hydrophobic tails anchoring within pocket-1 may also disfavor conformational transitions required for membrane fusion [22]. Downstream of entry, CBD’s PPARγ-dependent suppression of TLR4/NLRP3/Caspase-1 signaling in epithelial cells—with restoration of TEER and tight-junction proteins—offers a host-response rationale for the reduced interaction energies modeled for CBD-bound complexes, i.e., interface blockade may couple to mitigation of spike-triggered inflammatory signaling at mucosal surfaces [20]. Finally, CBD’s potent inhibition of M^pro^ (low-micromolar IC50) and moderate ACE2 inhibition—compared with weaker effects by Δ^9^-THC and lesser activity by CBN in those assays—are consistent with our observation that CBD maintains favorable binding across variants even when %SASA is lower in some contexts, underscoring the primacy of van der Waals/electrostatic complementarity over burial alone and suggesting potential synergy between entry-blocking and replication-stage inhibition within the same chemotype [23].

Although multiple 200 ns trajectories were generated for each system, the simulations primarily capture the comparative stability of the complexes within the sampled time window and may not fully reflect rare binding or unbinding events or large-scale conformational transitions. In addition, the simulations were performed using truncated ectodomain models without explicit glycans or full-length spike/ACE2 constructs, and the calculations relied on a single force field with defined assumptions regarding ion composition, which may influence the quantitative energy estimates. Finally, as the conclusions are derived from computational analyses, further biochemical and cellular experiments would be valuable to validate the predicted variant-spanning interface blockade by CBD and CBN.

## 3. Materials and Methods

### 3.1. Molecular Docking of Cannabis Compounds Targeting RBD/ACE2 Complexes

The WT RBD in complex with ACE2 (PDB ID 6M0J) was retrieved from the RCSB Protein Data Bank [33]. In addition, the Delta (PDB ID 7V8B) and Omicron BA.1 variant (PDB ID 7WBL) were retrieved from the RCSB Protein Data Bank [34,35]. Then, all ionizable amino acids were assigned protonation states at pH 7.0 using the PROPKA 3.0 web server (University of Copenhagen, Copenhagen, Denmark) [36]. Ligand protonation/ionization states at pH 7.0 were assigned prior to docking/MD simulation using ChemAxon Marvin v23.9 (ChemAxon, Budapest, Hungary). Neutral cannabinoids (e.g., CBD, CBN, THC-like scaffolds) were modeled in their neutral forms, whereas acidic cannabinoids (e.g., CBDA, CBGA, and THCA-A) were modeled in their deprotonated carboxylate forms. To block binding between RBD and ACE2, 11 cannabis compounds (Figure 2) were docked into the protein–protein interface of the WT RBD/ACE2 complex using the AutoDock VinaXB program (based on AutoDock Vina 1.1.2) [37], with a grid size of 40 Å × 50 Å × 30 Å, starting from the center of the RBD/ACE2 protein–protein interface. Subsequently, the lowest-energy complexes between drugs and complexes were selected for further study by MD simulations using the Amber20 program [38].

### 3.2. MD Simulations and Trajectory Analysis of RBD/ACE2-Cannabis Complexes

All parameters of WT RBD/ACE2 complexes docking with 11 cannabis compounds were prepared using the LEaP module in Amber20 [38]. All non-protein heteroatoms in the PDB files were removed prior to system preparation. Therefore, glycan moieties (if present in the deposited structures), crystallographic waters, ions, and other hetero groups were not explicitly modeled; only the protein chains were retained. The total charge of each system was neutralized by sodium ions. To simulate an explicit water system, the TIP3P water model was applied to solvate each complex in an octahedral periodic boundary water box with a distance of 12 Å from protein surfaces to the edge of the box [39]. After that, a 2 fs time step was used together with the SHAKE algorithm (ntc = 2, ntf = 2) to constrain all covalent bonds involving hydrogen atoms, while no additional restraints were applied to hydrogen-bond geometries during the production runs. In the relaxation step, the Langevin thermostat was used to control the temperature and pressure with a collisional frequency of 2 ps^−1^ and a pressure-relaxation time with the Berendsen barostat of 1 ps [40]. Then, the temperature of each simulated system was carefully heated from 100 to 310 K for 100 ps, while a harmonic constraint with 50.0 kcal/molÅ^2^ was applied to restrain all atoms of the protein. Before the production step, each system was equilibrated for 100 ps at 310 K with the same harmonic constraint in the heating step. Then, each system was simulated using the NPT ensemble at a temperature of 310 K and a pressure of 1 atm for 100 ns. Next, the structural trajectories of each system were analyzed using the CPPTRAJ module in AmberTools20 [41]. After the simulations were completed, the compounds that interacted with the binding pocket residues of the RBD/ACE2 complex were selected as candidate compounds to be docked and simulated with Delta and Omicron BA.1 RBD/ACE2 complexes, whose steps were similar to those in the WT systems. Then, the candidate compounds docking into the binding pocket of the WT, Delta, and Omicron BA.1 RBD/ACE2 were simulated up to 200 ns in three replicates. Finally, the three replicates of each system that consist of the same candidate compound within the same RBD/ACE2 complex were combined into one trajectory. For comparison, the apo-form WT, Delta, and Omicron BA.1 RBD/ACE2 complexes (no ligand) were also simulated using the same protocol for 200 ns in three independent replicas, enabling direct apo vs. ligand-bound comparisons in SIE and per-residue analyses.

The 1000 snapshots taken from the combined MD trajectories in the last 10 ns were analyzed using the CPPTRAJ module, including the root-mean-square deviation of atomic positions (RMSD), average solvent accessible surface area (SASA), and the percentage difference between the SASA of the system without compound (${SASA}_{Apo}$) and the SASA of the RBD/ACE2–cannabis complex (${SASA}_{Com}$), calculated following Equation (1):

(1) $$\%\mathrm{SASA}=(1-\frac{{SASA}_{Com}}{{SASA}_{Apo}})\times 100$$

The last 10 ns (190–200 ns) analysis window was selected because the protein and ligand RMSD values reached a plateau (Figure S2), indicating that the complexes had reached a stable regime suitable for detailed energetic and SASA analyses. Protein RMSD was calculated using all protein atoms after least-squares fitting, whereas ligand RMSD was calculated using heavy atoms after fitting on pocket-defining residues.

Pharmacophore models were generated using the LigandScout v4.4.2 (Inte:Ligand GmbH, Vienna, Austria) to represent the interactions between the candidate compound and the RBD/ACE2 complex. In addition, per-residue decomposition free energy (${\Delta G}_{bind}^{residue}$) and the binding free energies of protein–protein complexes (RBD and ACE2), as well as compound–protein interactions (candidate compound and RBD/ACE2 complex), were calculated using combined MD trajectories in the last 10 ns by employing MM/GBSA and SIE methods, respectively [27,42]. Moreover, to indicate the binding energy change (${\Delta \Delta G}_{bind}^{residue})$ of RBD and ACE2, the ${\Delta \Delta G}_{bind}^{residue}$ was estimated using Equation (2):

(2) $${\Delta \Delta G}_{bind}^{residue}={\Delta G}_{bind,Com}^{residue}-{\Delta G}_{bind,Apo}^{residue}$$

where ${\Delta G}_{bind,Com}^{residue}$ is ${\Delta G}_{bind}^{residue}$ of the compound blocking the RBD/ACE2 complex, and ${\Delta G}_{bind,Apo}^{residue}$ is ${\Delta G}_{bind}^{residue}$ of the apo-form system. In addition, the SIE calculation was used to predict the binding free energies between RBD and ACE2 and between the compound and RBD/ACE2 complex.

Solvated Interaction Energy (SIE) binding free energies ($∆G_{bind}$) were computed using the standard SIE parametrization:

(3) $$∆G_{bind}=\alpha (∆E_{vdW}+∆E_{c}+∆G^{R}+∆G^{cav})+\beta ,$$

where $∆E_{vdW}$ and $∆E_{c}$ are the Lennard–Jones and Coulomb interaction energies obtained from the AMBER force field, $∆G^{R}$ is the reaction-field solvation contribution, and $∆G^{cav}$ is a non-polar surface-area term. Energies were averaged over 1000 snapshots extracted every 10 ps from the 190–200 ns analysis window of the combined triplicate trajectories for each system [26,42].

Moreover, the residue interaction network methods were used to identify protein–protein connections at the atomic level [43,44]. The nodes (protein residues) and the edges (intra- and intermolecular interaction between protein chains) were generated from multiple 3D structures, and the network analysis techniques were applied to provide novel information on protein–protein interactions [43]. In this work, the RING was applied to identify the non-bonded interaction between the RBD and ACE2 from 200 snapshots taken from combined MD trajectories in the last 10 ns. In addition, the closest atom between two proteins was selected to calculate the nodes, and the multiple edges for the pair of nodes were established. In addition, distances between nodes were generated from strict distance thresholds using a default of the program, such as 3.5 Å for H-bonds, 4 Å for ionic bonds, 5 Å for π-cation, 0.5 Å for vdW, 6.5 Å for π-π stacking, and 2.5 Å for disulfide bonds. To demonstrate the network of the RBD and ACE2, the residues that had dc ≤ −2 kcal/mol were selected to investigate the pair of their residues and only intermolecular interactions that had >20% occupations were collected to generate the edges using the Cytoscape v3.9.1 (Cytoscape Consortium, San Diego, CA, USA) [43,44].

## 4. Conclusions

Computational screening of 11 cannabis compounds against the RBD/ACE2 interface identified CBD and CBN as the only ligands that consistently occupy pocket 1 over 200 ns MD in WT, Delta, and Omicron BA.1. CBN exhibited the most favorable ligand–complex binding in WT, whereas CBD retained favorable binding in Omicron BA.1 despite reduced interface burial, indicating that %SASA is not a reliable surrogate for affinity. Pharmacophore mapping and per-residue energy decomposition point to dominant van der Waals and hydrogen-bond contacts with key RBD (Y505 in WT/Delta; Y501 in Omicron BA.1) and ACE2 (E37, A387, R393) residues. Across systems, both ligands attenuated the modeled RBD–ACE2 binding, with CBD showing a slightly greater reduction than CBN. Overall, CBD and CBN emerge as promising in silico interface blockers for SARS-CoV-2 variants; follow-up biochemical and cellular assays are warranted to validate these predictions.

## Figures and Tables

**Table 1:** The binding free energy and interaction energy of the binding between CBD/CBN and RBD/ACE2, calculated using the SIE method.

Energy Component (kcal/mol)		CBD	CBN
$∆E_{vdW}$	WT	−32.84 ± 0.34	−40.01 ± 0.22
	Delta	−32.56 ± 0.60	−34.49 ± 0.28
	Omicron BA.1	−35.84 ± 1.00	−38.74 ± 0.34
$∆E_{c}$	WT	−8.59 ± 0.42	−4.61 ± 0.12
	Delta	−6.65 ± 0.33	−1.60 ± 0.11
	Omicron BA.1	−10.23 ± 0.77	−8.99 ± 0.26
$∆G^{R}$	WT	18.67 ± 0.26	15.90 ± 0.20
	Delta	17.53 ± 0.32	9.59 ± 0.17
	Omicron BA.1	24.81 ± 1.01	20.19 ± 0.34
∆MSAγ	WT	−7.79 ± 0.05	−7.51 ± 0.03
	Delta	−7.15 ± 0.11	−7.14 ± 0.05
	Omicron BA.1	−9.01 ± 0.14	−7.96 ± 0.06
$∆G_{bind}$	WT	−6.09 ± 0.08	−6.69 ± 0.03
	Delta	−5.91 ± 0.07	−6.41 ± 0.03
	Omicron BA.1	−6.06 ± 0.22	−6.61 ± 0.05

**Table 2:** Average SASA and %SASA of all systems. The SASA values were calculated from residues within binding sites between RBD and ACE2, while %SASA was calculated according to (1).

	∆SASA (Å^2^)			%SASA	
	Apo	CBD	CBN	CBD	CBN
WT	1517.87 ± 8.08	987.30 ± 7.89	727.26 ± 1.86	34.96	52.09
Delta	1381.10 ± 2.99	879.65 ± 3.28	904.08 ± 1.43	36.31	34.54
Omicron BA.1	1359.65 ± 13.55	997.64 ± 4.15	938.88 ± 2.37	26.63	30.95

**Table 3:** The SIE binding free energy and energy components of protein–protein binding between RBD and ACE2 with and without CBD or CBN bound.

Energy Component (kcal/mol)		Apo	CBD	CBN
$∆E_{vdW}$	WT	−92.52 ± 0.67	−92.07 ± 0.71	−91.17 ± 0.54
	Delta	−102.43 ± 0.59	−97.32 ± 0.72	−97.71 ± 0.62
	Omicron BA.1	−91.47 ± 0.51	−88.63 ± 0.56	−88.85 ± 0.55
$∆E_{c}$	WT	−286.96 ± 1.65	−264.98 ± 1.47	−271.50 ± 1.19
	Delta	−521.49 ± 2.98	−485.72 ± 2.70	−489.39 ± 1.86
	Omicron BA.1	−599.76 ± 1.73	−581.26 ± 1.62	−603.63 ± 2.46
$∆G^{R}$	WT	290.26 ± 1.42	293.62 ± 1.37	291.23 ± 1.28
	Delta	527.15 ± 3.00	490.97 ± 1.94	500.76 ± 1.42
	Omicron BA.1	600.66 ± 1.58	602.14 ± 1.58	621.45 ± 2.45
∆MSAγ	WT	−16.82 ± 0.10	−19.75 ± 0.12	−19.67 ± 0.08
	Delta	−19.01 ± 0.10	−16.99 ± 0.12	−17.01 ± 0.09
	Omicron BA.1	−16.64 ± 0.09	−18.89 ± 0.09	−19.22 ± 0.09
$∆G_{bind}$	WT	−14.00 ± 0.08	−11.60 ± 0.08	−12.43 ± 0.06
	Delta	−15.02 ± 0.09	−14.32 ± 0.13	−13.72 ± 0.11
	Omicron BA.1	−14.12 ± 0.07	−11.97 ± 0.08	−12.35 ± 0.09

## Data Availability

The data relevant to this study are available in the article and its Supplementary Materials. Additional simulation trajectories, as well as MM/GBSA and SIE analysis scripts, are available from the corresponding author upon reasonable request.

## Supplementary Materials

The following supporting information can be downloaded at: https://www.mdpi.com/article/10.3390/molecules31081253/s1, Figure S1. Distance between the centers of mass of the docking cannabis structure and the cannabis structure during 100–200 ns. Figure S2. RMSD time series from triplicate 200 ns molecular dynamics simulations of apo-, CBD-bound, and CBN-bound WT, Delta, and Omicron BA.1 RBD–ACE2 systems. Panels A–D show the RMSD profiles of the overall RBD–ACE2/ligand complex, RBD, ACE2, and the ligand, respectively. Rows correspond to WT, Delta, and Omicron BA.1, whereas columns correspond to apo, CBD, and CBN systems (panel D: CBD and CBN only). Blue, orange, and green traces indicate replicates 1, 2, and 3, respectively. Protein RMSD values were calculated on all protein atoms after least-squares fitting, whereas ligand RMSD values were calculated using heavy atoms after fitting on pocket-defining residues. Table S1. Definition of interface pockets and key pocket-defining residues at the RBD–ACE2 binding interface. Operational definitions and pocket-defining ACE2/RBD residues for pockets 1–3 used in this study. Table S2. Summary of molecular dynamics simulations performed in this study.
