# Supplementary material for: Molecular Dynamics and Solvated Interaction Energy Prioritize Cannabidiol and Cannabinol as Variant-Spanning SARS-CoV-2 RBD–ACE2 Interface Blockers
Source: Molecules. 2026 Apr 10;31(8):1253. doi: 10.3390/molecules31081253 (PMC13118358; doi:10.3390/molecules31081253)
Supplement: Supplementary file 1 [file molecules-31-01253-s001.zip › molecules-4123621-supplementary.pdf]

Supplementary

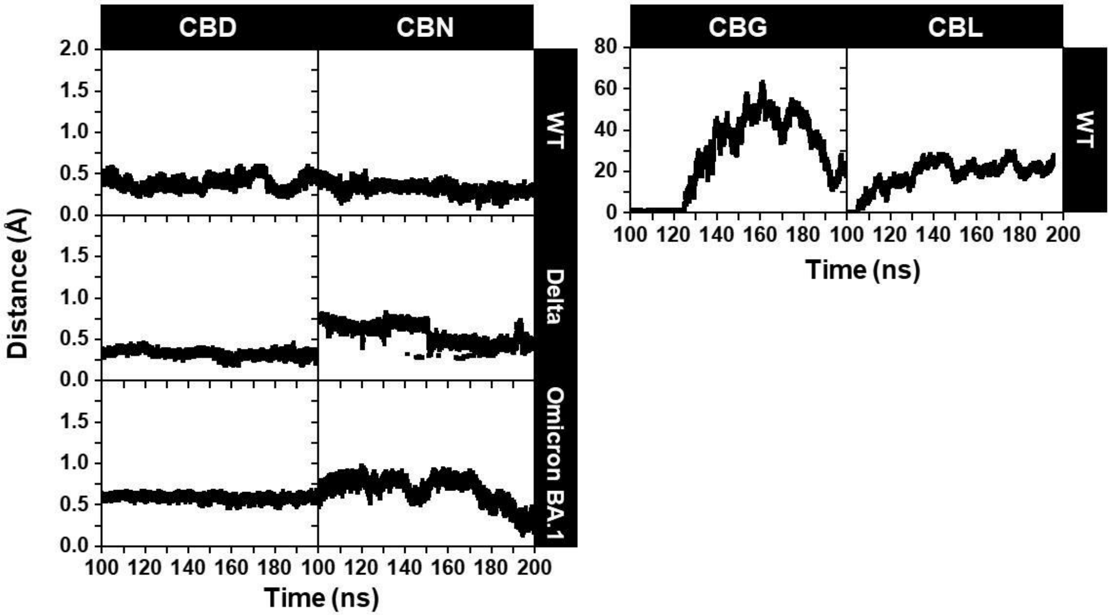

Figure S1. Distance between the centers of mass of the docking cannabis structure and the cannabis structure during 100–200 ns.

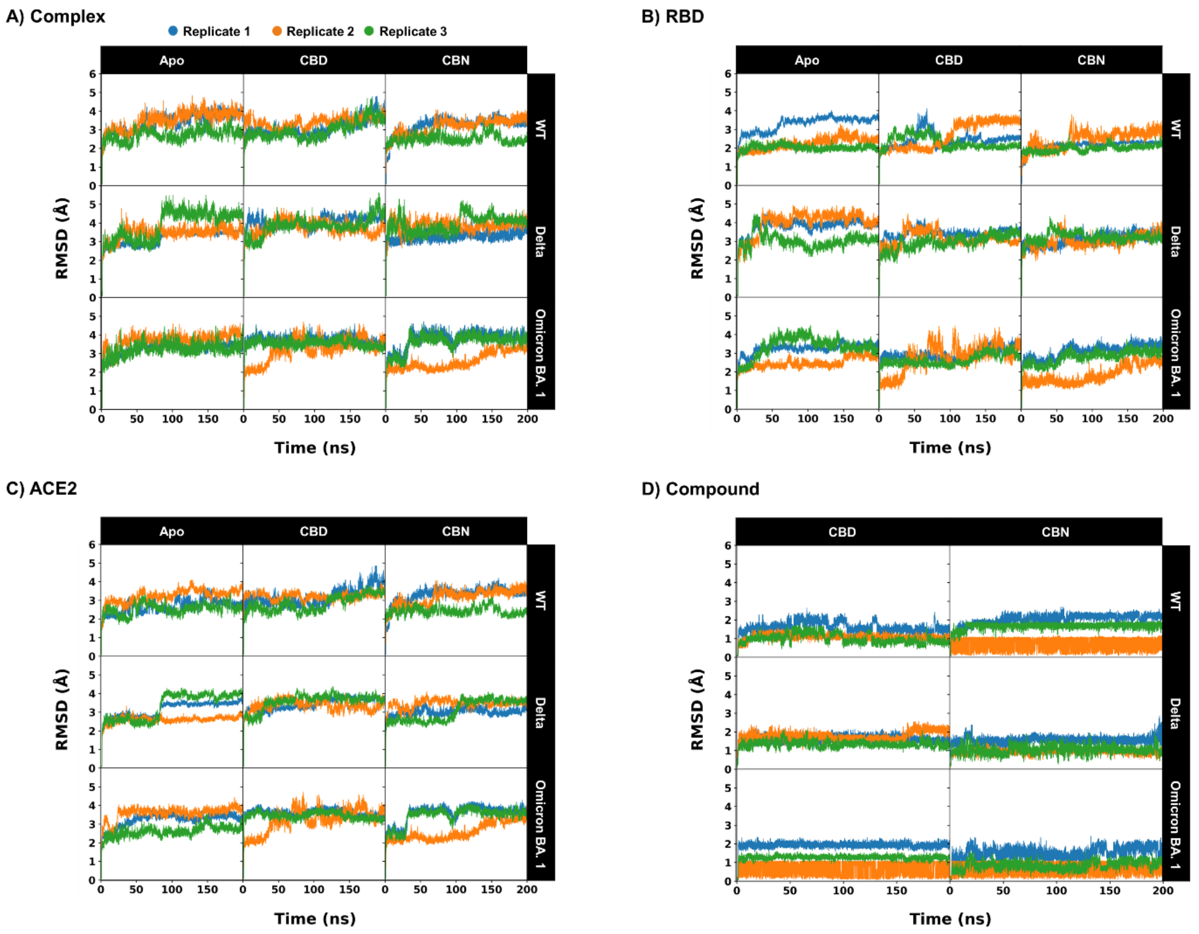

Figure S2. RMSD time series from triplicate 200 ns molecular dynamics simulations of apo-, CBD-bound, and CBN-bound WT, Delta, and Omicron BA.1 RBD–ACE2 systems. Panels A–D show the RMSD profiles of the overall RBD–ACE2/ligand complex, RBD, ACE2, and the ligand, respectively. Rows correspond to WT, Delta, and Omicron BA.1, whereas columns correspond to apo, CBD, and CBN systems (panel D: CBD and CBN only). Blue, orange, and green traces indicate replicates 1, 2, and 3, respectively. Protein RMSD values were calculated on all protein atoms after least-squares fitting, whereas ligand RMSD values were calculated using heavy atoms after fitting on pocket-defining residues.

Table S1. Definition of interface pockets and key pocket-defining residues at the RBD–ACE2 binding interface. Operational definitions and pocket-defining ACE2/RBD residues for pockets 1–3 used in this study.

Pocket	Operational definition	ACE2 residues	RBD residues
**Pocket 1**	Cavity around the **Y505 / N501(Y)** hot spot where CBD/CBN persist across variants	N33, H34, E37, D38, A387, Q388, P389, R393, K353	R403, D405/E406, Y453, Y495, F497, **Q498/R498, N501/Y501, Y505/H505**
**Pocket 2**	Patch near the ACE2 N-terminal helix; enriched in polar/salt-bridge contacts at the interface [40]	D30, K31, E35, Y41, Q42	**K417/N417**, L455, F456, **Q493/K493**, **Q498/R498**
**Pocket 3**	Hydrophobic subpocket accommodating RBD hydrophobic residues (e.g., F486) via ACE2 hydrophobic cluster [40]	F28, L79, M82, Y83	F486, N487, Y489

Table S2 Summary of molecular dynamics simulations performed in this study. (Systems, ligands, production length, number of replicas, and analysis notes.)

**Stage**	**System (protein complex)**	**Ligand(s)**	**Production length (ns)**	**Replicas**	**Notes/output used**
Screening MD	WT RBD/ACE2	11 cannabis compounds (Fig. 2)	100	1 each	Used to assess ligand stability; CBD/CBN selected
Focused MD	WT RBD/ACE2	CBD	200	3	Replicas combined; last 10 ns used for analyses
Focused MD	WT RBD/ACE2	CBN	200	3	Replicas combined; last 10 ns used for analyses
Focused MD	Delta RBD/ACE2	CBD	200	3	Replicas combined; last 10 ns used for analyses
Focused MD	Delta RBD/ACE2	CBN	200	3	Replicas combined; last 10 ns used for analyses
Focused MD	Omicron BA.1 RBD/ACE2	CBD	200	3	Replicas combined; last 10 ns used for analyses
Focused MD	Omicron BA.1 RBD/ACE2	CBN	200	3	Replicas combined; last 10 ns used for analyses
Apo control	WT/Delta/ Omicron BA.1 RBD/ACE2	none	200	3 each	Used for protein–protein binding comparison (Table 3)
